# Using Smartbands, Pupillometry and Body Motion to Detect Discomfort in Automated Driving

**DOI:** 10.3389/fnhum.2018.00338

**Published:** 2018-09-24

**Authors:** Matthias Beggiato, Franziska Hartwich, Josef Krems

**Affiliations:** Department of Psychology, Cognitive and Engineering Psychology, Chemnitz University of Technology, Chemnitz, Germany

**Keywords:** discomfort, automated driving, smartband, pupillometry, psychophysiology, motion tracking

## Abstract

As technological advances lead to rapid progress in driving automation, human-machine interaction (HMI) issues such as comfort in automated driving gain increasing attention. The research project KomfoPilot at Chemnitz University of Technology aims to assess discomfort in automated driving using physiological parameters from commercially available smartbands, pupillometry and body motion. Detected discomfort should subsequently be used to adapt driving parameters as well as information presentation and prevent potentially safety-critical take-over situations. In an empirical driving simulator study, 40 participants from 25 years to 84 years old experienced two highly automated drives with three potentially critical and discomfort-inducing approaching situations in each trip. The ego car drove in a highly automated mode at 100 km/h and approached a truck driving ahead with a constant speed of 80 km/h. Automated braking started very late at a distance of 9 m, reaching a minimum of 4.2 m. Perceived discomfort was assessed continuously using a handset control. Physiological parameters were measured by the smartband Microsoft Band 2 and included heart rate (HR), heart rate variability (HRV) and skin conductance level (SCL). Eye tracking glasses recorded pupil diameter and eye blink frequency; body motion was captured by a motion tracking system and a seat pressure mat. Trends of all parameters were analyzed 10 s before, during and 10 s after reported discomfort to check for overall parameter relevance, direction and strength of effects; timings of increase/decrease; variability as well as filtering, standardization and artifact removal strategies to increase the signal-to-noise ratio. Results showed a reduced eye blink rate during discomfort as well as pupil dilation, also after correcting for ambient light influence. Contrary to expectations, HR decreased significantly during discomfort periods, whereas HRV diminished as expected. No effects could be observed for SCL. Body motion showed the expected pushback movement during the close approach situation. Overall, besides SCL, all other parameters showed changes associated with discomfort indicated by the handset control. The results serve as a basis for designing and configuring a real-time discomfort detection algorithm that will be implemented in the driving simulator and validated in subsequent studies.

## Introduction

Automated driving is expected to bring several mobility benefits such as improved traffic safety, reduced congestions and emissions, social inclusion, accessibility and more comfort (ERTRAC, 2017). As technological advances have enabled the rapid progression in driving automation, human-machine interaction (HMI) issues gain more attention and are considered a key question for broad public acceptance (Banks and Stanton, 2016; Riener et al., 2016; ERTRAC, 2017). One central HMI issue involves the question of how comfortable automated driving can be implemented to ensure a positive driving experience (Elbanhawi et al., 2015; ERTRAC, 2017; Bellem et al., 2018). Having a positive driving experience is a main factor for deciding to purchase and use a vehicle or in-vehicle system (Engelbrecht, 2013). In automated driving, discomfort could additionally lead to potential safety-critical situations, for example, due to (non-necessary) takeover with all associated risks such as reduced situation awareness (Hergeth et al., 2017). As the human role in automated driving changes from active driver to passenger, new and additional determinants of driving comfort are discussed, such as motion sickness, apparent safety, trust in the system, feelings of control, familiarity of driving maneuvers, and information about system states and actions (Beggiato et al., 2015; Elbanhawi et al., 2015; Bellem et al., 2016). There is no agreed-upon definition for comfort in the scientific community (Hartwich et al., 2018); however, existing comfort definitions share some central assumptions: comfort (a) is a subjective construct and, therefore, differs between individuals; (b) is affected by physical, physiological, and psychological factors; and (c) results from interaction with the environment (de Looze et al., 2003). Thus, comfort is hereby understood as a subjective, pleasant state of relaxation expressed through confidence and apparently safe vehicle operation (Constantin et al., 2014), “which is achieved by the removal or absence of uneasiness and distress” (Bellem et al., 2016, p. 45).

The research project KomfoPilot at Chemnitz University of Technology aims to investigate factors that influence comfort in automated driving. One objective is to find parameters that affect comfort on a general level, for example, situations and driving parameters such as speed, longitudinal/lateral distance, driving style familiarity, or personal characteristics (Hartwich et al., 2015, 2018). A second objective is the development of an algorithm for real-time discomfort detection to adapt driving style and information presentation at each moment once discomfort begins. The underlying idea is the metaphor of a vehicle–driver–team that knows each other’s strengths, limitations, and current states, and is able to react accordingly (Klein et al., 2004). The algorithm will be developed by project partners who specialize in data fusion (FusionSystems GmbH and Communication Engineering Department at Chemnitz University of Technology) and should combine data from different sensors such as in-car sensors (2D and 3D cameras, motion tracking), physiological sensors (smartband Microsoft Band 2, eye tracking), vehicle data and environment sensors. As a basis for developing the algorithm, the present article reports the results of the psychophysiological parameters pupil diameter, eye blink frequency, heart rate (HR), heart rate variability (HRV), electrodermal activity (EDA) and body motion with regard to discomfort during automated driving in a driving simulator. Driving simulators offer an optimal environment for creating standardized situations under experimental control and applying sensors for measuring physiological parameters (Brookhuis and de Waard, 2011), although with limited external validity. The presented analyses aim to provide information about the potential of each parameter for detecting discomfort in an approaching automated situation, such as overall relevance, variability, direction and strength of effects, timing such as increase and decrease before and after discomfort as well as filtering and artifact removal strategies.

The use of these physiological parameters to infer mental states has a long research tradition. Despite results that are often contradictory, the main findings for these parameters are summarized subsequently and hypotheses regarding discomfort are derived. Pupil diameter has been studied largely as an indicator for mental effort, cognitive workload, stress, fatigue, information processing, affective processing and attention (Andreassi, 2000; Cowley et al., 2016). One of the major challenges in interpreting pupil size changes out of controlled lab studies is the heavy dependance on ambient light (Palinko and Kun, 2012). Despite these problems in separating the effects of ambient factors and mental states, a central finding is that pupil diameter increases with task difficulty, mental workload, emotionality of stimuli, and information-processing demands (Andreassi, 2000; Backs and Boucsein, 2000; Cowley et al., 2016). Thus, an increase in pupil diameter is expected during uncomfortable situations. Eye blink rate is considered a sensitive indicator for mental workload, mood states, fatigue and task demands (Andreassi, 2000; Cowley et al., 2016). A decrease in blink rate in complex situations requiring visual attention has been found for car driving in complex situations as well as for fighter pilots (Backs and Boucsein, 2000). Thus, a decrease in eye blink rate is expected during discomfort situations in automated driving, which are visually monitored by the driver.

The cardiovascular parameters HR and HRV are often used in driving simulation and on-road driving studies as indicators of mental effort, stress, workload, and task demands (see the overview of studies in Backs and Boucsein, 2000; Mulder et al., 2005; Brookhuis and de Waard, 2011; Mehler et al., 2012; Ahonen et al., 2016; Schmidt et al., 2016). A common finding is that with higher invested effort and stress, HR increases and HRV decreases. The discomfort-inducing close approach situation investigated in this study could be seen as analogous to stress situations, including the uncertainty about the capability of a system to successfully complete a task. Thus, an increase in HR and a decrease in HRV during uncomfortable situations are expected. Similar to HR and HRV, EDA has a long tradition in psychophysiological research. Common findings include an increase of skin conductance level (SCL) with higher arousal, alertness, mental effort, workload, emotional load, stress, and task difficulty (Dawson et al., 2017). However, as EDA is sensitive to a wide variety of stimuli, it is not a clearly interpretable measure of any particular psychological process and must be interpreted by including the stimulus conditions (Cowley et al., 2016; Dawson et al., 2017). For discomfort, an increase in SCL is expected due to a prediction of higher alertness and arousal. The HR, HRV, and EDA were measured using the smartband Microsoft (MS) Band 2. The use of a commercially available smartband was an explicit project goal to estimate the potential and problems of such a psychophysiological sensor. On the one hand, the market for smartbands is growing (Wade, 2017); thus, smartbands connected to vehicles could be an option for assessing psychophysiological parameters inside cars. On the other hand, the MS Band 2 has already been used in research for assessing mental workload in different environments (Binsch et al., 2016; Cropley et al., 2017; Reinerman-Jones et al., 2017; Schmalfuß et al., 2018), activity recognition in a home setting (Filippoupolitis et al., 2016), and for predicting and regulating personal thermal comfort in buildings (Laftchiev and Nikovski, 2016; Li et al., 2017).

Body motion during driving has mainly been investigated with regard to head movements for predicting driver intentions (Pech et al., 2014), hand movements for estimating driver distraction (Tran and Trivedi, 2009), trapezius muscle tension as an indicator for stress (Morris et al., 2017), or facial features for monitoring driver states (Baker et al., 2004). Moreover, the whole 3D driver posture is considered potentially useful for extracting information related to intentions, affective states, and distraction (Tran and Trivedi, 2010). However, posture dynamics are strongly related to situations and should, therefore, be combined with other contextual information (Tran and Trivedi, 2010). In the specific approach situation with the danger of a potential rear-end collision, a pushback movement is expected that should be reflected in motion tracking and seat pressure mat data. Table 1 provides a summary of the expected effects during discomfort periods for all parameters.

**Table 1:** Overview of expected effects of different sensor parameters during discomfort.

Sensor	Parameter	Expected trend during discomfort
Eye tracking	Pupil diameter	Dilation (increase of diameter during mental effort/stress/attention/task difficulty)
	Blink rate	Decrease (attention/arousal/alertness)
MS Band 2	Heart rate	Increase (mental workload/stress)
	Heart rate variability	Decrease (mental workload/stress)
	Skin conductance level	Increase (mental workload/arousal/alertness/emotional response/stress)
Motion tracking	Shoulder/head movements	Push-back/lean-back (decrease on z-axis)
Pressure mat	Pressure	Push-back/lean-back (pressure increase at back position sensor)

## Materials and Methods

### Study Design and Route

**Figure 1:**
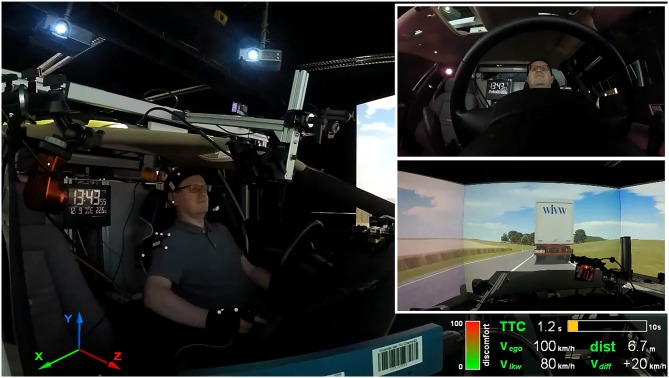
Setup of the driving simulator study during one approach situation to the truck driving ahead. Left side: motion capture markers at head, right shoulder, and hands; handset control for reporting discomfort held in the right hand. Right side: driver camera view (top); front scenery camera view recorded from the roof of the mock-up (middle); reported discomfort as well as driving parameters at that particular moment such as TTC, ego speed/truck speed/speed difference and distance to the truck (bottom). Written informed consent was obtained from the individual for the publication of this image.

**Figure 2:**
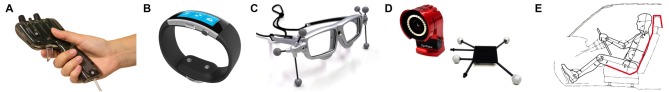
Sensors: **(A)** Handset control for reporting discomfort; **(B)** smartband Microsoft Band 2; **(C)** SMI Eye Tracking Glasses 2; **(D)** camera and rigid body for motion tracking; **(E)** schematic layout and placement of seat pressure mat.

The driving simulator study was composed of two separate driving sessions with an approximate 2-month delay in between. Every driving session was composed of a 3-min highly automated trip on a straight, single carriageway, rural road. The trip was prerecorded and was exactly the same for all participants; there was no possibility to intervene by pedals or steering wheel. In every session, participants experienced three identical and potentially discomfort-inducing approach situations with the danger of a potential rear-end collision (Figure 1). A white truck drove in front of the ego car with a constant speed of 80 km/h, whereas the ego car approached in a fully automated mode at 100 km/h. Automated braking was initialized very late at a distance of 9 m, which resulted in a minimum distance of 4.2 m and minimum time to contact of 1.1 s. After the approach, the ego car fell back at a distance of 100 m, and the approach started again. Participants were not informed about the situation and were instructed to press the lever of the handset control (Figure 2A) according to the extent of perceived discomfort. Thus, every participant experienced six approach situations in total, which resulted in 240 situations for all 40 participants and both sessions. The main reasons for inviting the participants twice were to: (a) obtain a higher overall number of discomfort situations per person; and (b) assess habituation effects within subjects over short and longer time periods (3 min vs. 2 months). Evaluation of habituation effects resulted in small to almost no effects, both for short- and long-term periods. Thus, all situations were included in the subsequent analyses.

#### Participants

A total of 40 participants (15 females, 25 males) took part in both sessions of the study. Ages ranged from 25 years to 84 years with two distinct age groups, one from 25 years to 45 years (younger group, *N* = 21, *M* = 30 years, *SD* = 4.3) and the other over 65 years (older group, *N* = 19, *M* = 72 years, *SD* = 6.0). All subjects were required to currently hold a valid driver’s license, and none of them had had previous experience of highly automated driving in the driving simulator. Participants were compensated with 20 euros for participation. This study was carried out in accordance with the recommendations, regulations and consent templates of the TU Chemnitz ethics commission. The protocol was approved by the TU Chemnitz ethics commission. All subjects gave written informed consent in accordance with the Declaration of Helsinki.

#### Material and Sensors

The study took place in a fixed-base driving simulator (SILAB 5.1 Software) with a fully equipped interior, a rear-view mirror, two side mirrors and a 180° horizontal field of view. Fully automated trips were prerecorded and replayed, while the participants sat in the driver’s seat. Pedals and steering wheel were inoperative during these trips. Perceived discomfort was assessed during the whole trip by a handset control integrated into the driving simulator (Hartwich et al., 2015, 2018; Figure 2A). Participants could press the lever gradually in accordance with the extent of perceived discomfort. The smartband Microsoft Band 2 (Figure 2B) was used to record the physiological parameters of HR, HRV and SCL via a Bluetooth connection. Accelerometer and gyroscope data were recorded as well from the band sensors to identify and correct for hand movements. The MS Band 2 was provided with a Software Development Kit that allowed for programming a dedicated logging application. Eye tracking data were recorded by SMI Eye Tracking Glasses 2 (SMI ETG 2, Figure 2C) and included pupil diameter, fixations, saccades and blinks. Participants already wearing eyeglasses (*N* = 10) could not wear the SMI ETG 2, which resulted in less eye tracking data. In addition, the SMI ETG 2 were not applied in the whole second driving session because of testing camera-based, facial-feature recognition algorithms. Body motion was simultaneously captured by two sensor systems. The first device was a marker-based motion tracking system from OptiTrack composed of four Flex 13 infrared cameras recording with 120 fps (Figure 2D). A total of four distinct rigid bodies were tracked (left and right hand, right shoulder and head; see Figures 1, 2D). Rigid bodies are a collection of three or more markers on an undeformable object. These rigid bodies can be attached to tracked objects (e.g., clothes, gloves, headbands) and allow for recording position and orientation in six degrees of freedom. Participants with eyeglasses wore a headband with the rigid body attached (as in Figure 1), whereas the SMI ETG 2 allowed for directly attaching rigid bodies (Figure 2C). The second sensor system for body motion was a seat pressure mat developed by the project partner FusionSystems GmbH (Figure 2E). The mat can easily be placed on top of the seat and includes eight pressure sensors at different positions.

### Data Recording and Sequence Extraction

**Figure 3:**
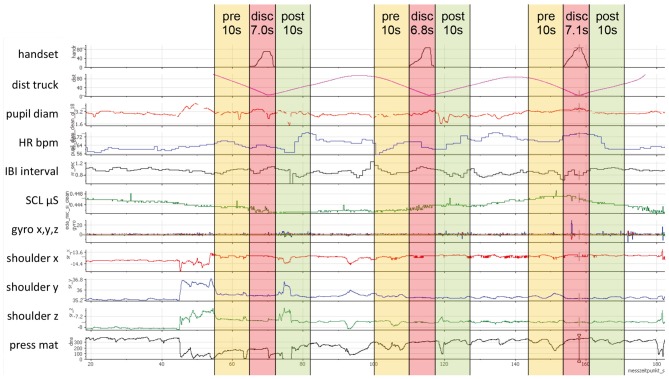
Example of synchronized sensor data during one trip, handset values in the first sensor channel and three extracted discomfort intervals (disc) with 10 s before (pre) and 10 s after (post).

Data were recorded by several independent data loggers for each sensor with different recording frequencies. System time for all recording devices was continuously synchronized with a software tool based on the network time protocol (Meinberg NTP Software). Recording frequencies were 60 Hz for the driving simulator data, including handset control, 10 Hz for the MS Band 2, 60 Hz for the SMI ETG 2 eye tracking data, 120 Hz for motion tracking, and 10 Hz for the seat pressure mat. Raw data for each recorder were imported into a storage and analysis framework based on the relational open-source database management system PostgreSQL (Beggiato, 2015). The synchronization procedure was based on the timestamps of the driving simulator data (60 Hz) by adding the current value of all other sensor systems at this specific moment. To analyze changes in the sensor data with regard to perceived discomfort, data during reported discomfort by the handset control were compared with 10-s time intervals prior and after (Figure 3).

**Figure 4:**
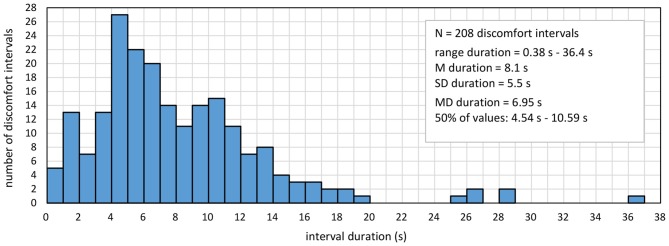
Distribution and descriptive statistics of the 208 extracted discomfort intervals.

Discomfort intervals were extracted from the start of pressing the handset control lever until releasing, independent of the magnitude. However, the handset control was only pressed in 208 of the 240 approach situations. The distribution and descriptive statistics of the 208 extracted discomfort intervals are presented in Figure 4. In addition, single sensor channels were not recorded in some situations (e.g., no SMI ETG 2 for subjects already wearing eyeglasses or technical problems). Thus, all charts in the results section contain the respective number and mean duration of discomfort intervals that were included in the analysis. For the subsequent results section, the term “sequence” refers to the whole time period including the discomfort interval as well as the 10 s beforehand and afterwards.

### Data Preparation

#### Common X-Axis

**Figure 5:**
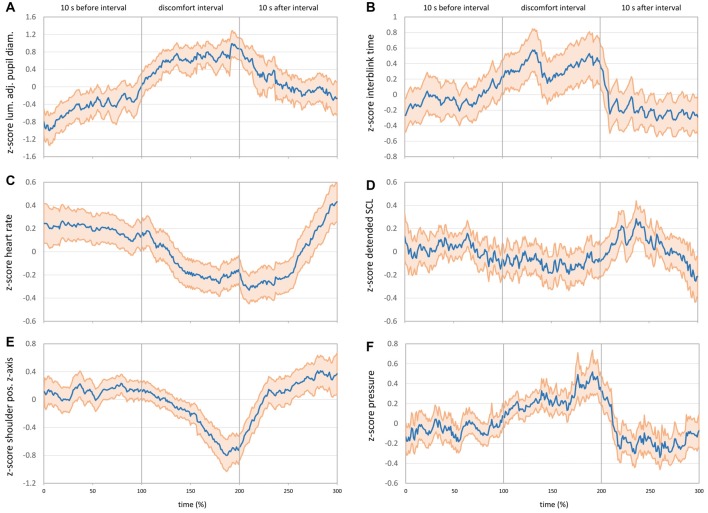
**(A)** Mean luminance-adjusted *z*-score of pupil diameter before, during and after discomfort intervals (*N* = 65 intervals, *M* = 7.65 s, *SD* = 4.74 s). **(B)** Mean *z*-score of interblink interval time before, during and after discomfort intervals (*N* = 67 intervals, *M* = 7.73 s, *SD* = 4.70 s). **(C)** Mean *z*-score of HR before, during and after discomfort intervals (*N* = 206 intervals, *M* = 8.10 s, *SD* = 5.52 s). **(D)** Mean *z*-score of detrended SCL before, during and after discomfort intervals (*N* = 203 intervals, *M* = 8.16 s, *SD* = 5.51 s). **(E)** Mean *z*-score of right shoulder movements on the z-axis before, during and after discomfort intervals (*N* = 114 intervals, *M* = 7.85 s, *SD* = 4.79 s). **(F)** Mean *z*-score of pressure mat sensor at the back position before, during and after discomfort intervals (*N* = 202 intervals, *M* = 8.04 s, *SD* = 5.41 s). The bold blue line shows the mean values, and the light red area shows the 95% pointwise confidence interval (CI) in all charts.

To show the development of all assessed parameters before, during, and after the discomfort interval, a common time axis was created for the charts in the results section (Figure 5). As the discomfort intervals varied in duration (Figure 4), a percent scale from 0% to 300% over the whole sequence was used to allow for displaying all values in the same scale. Periods before and after the discomfort interval were always 10 s long; thus, 1% corresponds to 0.1 s. Each discomfort interval was divided into percent slices, and the mean of each parameter was calculated for the specific time period of the respective percent slice. Finally, each percentage section before, during, and after reported discomfort was combined into one chart to show the progress of values over time. As not every sensor was active during the trips, each chart contains the number of sequences with mean duration and standard deviation of the included discomfort intervals in the caption. The main reason for using the percentage scale was to strictly respect the subjective aspect of discomfort mentioned in the definition. Thus, the different durations of reported discomfort intervals should enter with the same weight in all analyses, which can be obtained by the percentage scale. In addition, the analysis method should also be applicable in less standardized situations, which requires a reliance on the reported handset values. However, using the percentage scale also has some drawbacks. It is not possible to give precise time-related indications, as it is not time, but the subjectively reported intervals that represent the unit of measurement. However, descriptive statistics about the intervals presented in Figure 4 provide an indication of temporal dimensions. A second drawback is in regard to short sequences of a few seconds, in which some physiological processes such as changes in HR and SCL could hardly take effect. Similar concerns could be raised regarding longer sequences in terms of outliers, such as the six sequences over 20 s (Figure 4). However, as the percentage scale assigns the same weight to all sequences, excluding these six sequences does not change the results (tested for all analyses). Thus, despite these mentioned potential drawbacks, all sequences were included to present the overall picture.

#### Z-Standardization and 95% Confidence Intervals

An important issue in processing psychophysiological data is distinguishing the signal of interest from noise (Gratton and Fabiani, 2017). Most of the physiological parameters such as HR or EDA have a strong individual component, which means that absolute values can hardly be compared between subjects. Thus, relative changes within one person provide better signal-to-noise ratio, for example, comparing changes of HR or EDA before, during and after discomfort intervals. However, these changes need to be transformed into a common scale to be compared between subjects. One of the common and best-performing transformations is the *z*-score, which expresses all values as the distance to the mean in units of standard deviations with a total mean of zero and a standard deviation of one (Jennings and Allen, 2017). *Z*-transformation was applied for each sequence, resulting in the relative changes over time in units of standard deviations. Resulting *z*-values were averaged over all sequences at each single percent level from 0% to 300% and displayed as a blue line in the results charts (Figure 5). Beside these general transformations, some parameter-specific data correction methods and transformations were applied and are described for each parameter in the subsequent results sections. To obtain a quick estimation about the statistical significance of changes over time, the 95% confidence interval (CI) of each of these means was calculated pointwise and plotted as a light red area around the blue means. If the 95% CI does not overlap between two points in time, these two means differ in a statistically significant manner at *p* < 0.01 (Field, 2013). The pointwise CI does not include multiplicity correction as would be the case for simultaneous confidence bands. Simultaneous CI bands control for the familywise error in autocorrelated time series by estimating the simultaneous coverage probability of the whole curve (Korpela et al., 2014; Francisco-Fernández and Quintela-del-Río, 2016; Ahonen et al., 2018). As the aim of the present analyses is not to fit a curve, but allow for visual comparison of single points in time, pointwise CIs were used. Pointwise CIs are narrower than a simultaneous CI band would be, and pointwise CIs allow only for comparing single points (as an ANOVA would do), but do not appropriately reflect the CI for the curve as a whole.

## Results

### Pupil Diameter and Eye Blinks

Raw pupil diameters for the left and right eye (mm) from the SMI ETG 2 were averaged to get a single diameter from both eyes. To correct for signal fluctuations (especially close to blinks), a moving average over ±300 ms was calculated, and a *z*-transformation of these values was applied for each sequence. As pupil diameter is not only dependent on mental states, but primarily on ambient light (Watson and Yellott, 2012), the metric could potentially be confounded during the white truck approach situation. Thus, the mean luminance value of all pixels (HSL color model) was calculated for each video frame of the SMI ETG 2 front camera video. A *z*-transformation of this mean luminance was applied for the whole trip in order to subtract these luminance *z-scores* from the *z-scores* of pupil diameter. The resulting luminance-adjusted *z-values* of pupil diameter are shown in Figure 5A. In line with the hypotheses, pupil diameter increased significantly during the discomfort interval and decreased steadily after reported discomfort. About 5 s after the end of the discomfort interval (approx. 250%), the 95% CI does not overlap anymore with the 95% CI during the discomfort interval (side note: without correcting for ambient luminance, the effects are the same but more pronounced).

**Figure 6:**
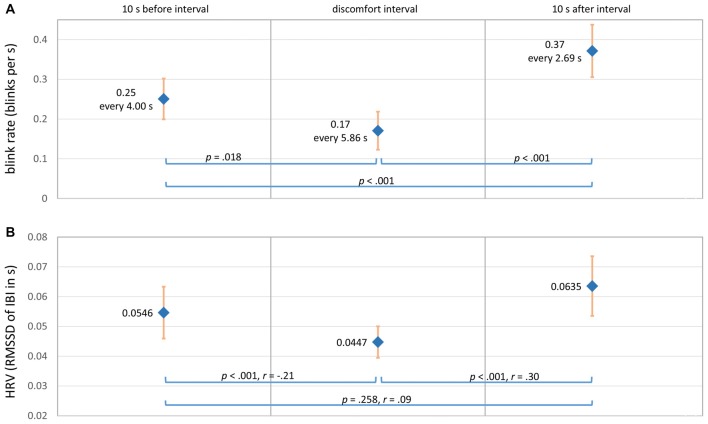
**(A)** Mean eye blink rate before, during and after discomfort intervals (*N* = 67 intervals, *M* = 7.73 s, *SD* = 4.70 s). **(B)** Mean HRV (RMSSD) before, during and after discomfort intervals (*N* = 202 intervals, *M* = 8.10 s, *SD* = 5.53 s). The bold blue dots show the mean values, and the light red bars show the 95% pointwise CI.

Eye blink rate recorded by the SMI ETG 2 was computed in two different ways: first, blinks per second were calculated for each whole interval before, during, and after reported discomfort. Figure 6A shows the expected decrease in blink rate from 0.25 blinks per second before discomfort to 0.17 blinks per second during discomfort and the increase afterwards to 0.37 blinks per second (*F*_(1.37,118.57)_ = 26.37, *p* < 0.001, $\eta_{\text{p}}^{2}$ = 0.285). However, this representation of blink rate does not allow for judging timings of increase/decrease as well as significance levels over time. Thus, it does not provide information for parameterizing an online detection algorithm. Therefore, a second way of obtaining a continuous blink rate was applied by calculating a running “interblink interval time.” This timer is set to zero every time a new blink is detected by the eye tracker and increases until the subsequent eye blink start is detected. Blink duration is not excluded and enters the running time. *Z*-values of this running interblink interval time were calculated for each sequence and averaged for each percent of time. Figure 5B shows the progress of interblink interval time *z*-scores with a noticeable increase during discomfort intervals (meaning less blinks) and the return to the prior level after the discomfort interval.

### Heart Rate and Heart Rate Variability

Raw HR values in beats per minute recorded by the MS Band 2 were transformed into *z*-values for each of the 206 sequences. Figure 5C shows the mean *z*-scores for HR over time. In contrast to the hypothesis, HR decreased steadily at the beginning of the discomfort interval. The bottom HR plateau was reached at about the middle of the discomfort interval (150%) and kept until about 5 s after reported discomfort (250%). Afterward, HR rapidly rose up to approximately the prior level.

The HRV was computed using the interbeat interval times (IBI) in s from the MS Band 2. The HR and IBI are not exact reciprocal values in the case of the MS Band 2, but IBI is recommended for HRV calculations (Cropley et al., 2017). The time-domain metric root mean square successive difference (RMSSD) was calculated for each interval and averaged over all 202 sequences. The RMSSD is recommended for measuring high-frequency HRV and when time intervals to compare are not equally long (Berntson et al., 2017). Frequency domain and nonlinear HRV measures were not applied due to the relatively short time periods investigated. In line with the hypothesis, Figure 6B shows the expected u-shaped pattern with a decrease of HRV during reported discomfort ($\chi_{\left(2\right)}^{2}$ = 40.05, *p* < 0.001; nonparametric Friedman’s ANOVA).

### Skin Conductance Level

Two electrodes on the opposite side of the MS Band 2 display (Figure 2B) measured skin resistance level in kilo ohm. These values were inverted and multiplied by 1,000 to obtain the SCL in micro Siemens. The SCL values were very sensitive to changes in the hand/arm position such as placing a hand on the knees. Thus, SCL values were excluded (missing data) during high-movement episodes on the basis of the MS Band 2 accelerometer and gyroscope data. The remaining values were *z*-standardized for each sequence. Results showed a continuous linear increase of SCL over time, independent of the situation. As this linear growing trend was probably related to the fact that subjects simply got warm during driving, a detrending algorithm was applied. Thus, a linear regression was calculated for each sequence. The SCL *z*-scores were subtracted from the regression values in order to obtain detrended *z*-scores, which are shown in Figure 5D. Detrended SCL showed almost no changes during the discomfort interval compared with the interval before and after.

### Body Movements

To assess body movements, data from the marker-based motion tracking system as well as the seat pressure mat were evaluated. The position of the right shoulder (mm) was captured by the motion tracking system. As the absolute marker position in the 3D space differed for each individual subject and each drive, differences on the z-axis position were computed for each sequence starting with zero at the beginning of the sequence. These value changes were transformed into *z*-scores. Figure 5E shows the mean *z*-scores of shoulder movement on the z-axis. As expected, the pushback of the body was represented by the u-shaped decrease of the shoulder *z*-position during the discomfort interval. Shoulder movements on the *x*- and *y*-axis showed similar but weaker effects; the main movement was backwards.

The pushback movement should also be represented in the data of the seat pressure mat, which would potentially allow for an easier movement measurement than motion tracking. To analyze the seat pressure mat data, the sensor at the back position was taken into account. Pressure values were *z*-transformed for each sequence. The *z*-scores of the pressure sensor (Figure 5F) showed the corresponding pattern to the motion-tracking results with an increase of pressure during the discomfort interval (pushback movement).

## Discussion

The present study aimed at detecting discomfort in automated driving by physiological parameters from smartbands, pupillometry and body motion. Discomfort is considered an important issue for broad public acceptance of automated vehicles as well as for safety issues such as critical and not-necessary take-over situations. Considering the metaphor of a vehicle-driver-team that knows each other, automated systems could react to detected discomfort by changing driving style parameters and information presentation. An important basis for a real-time discomfort detection algorithm is information about physiological sensor parameters associated with reported discomfort, such as overall relevance, direction and strength of effects, timings, variability as well as filtering and artifact removal strategies.

Overall, besides SCL, all other assessed parameters like pupil diameter, eye blink rate, HR, HRV and body motion showed changes associated with discomfort indicated by the handset control. However, filtering and standardization procedures are required to increase the signal-to-noise ratio and remove bias caused by individual differences. In addition, every parameter has its own specificities, which are subsequently discussed.

Pupil diameter showed the expected inverse u-shaped pattern with a dilation during discomfort and recovery afterward, analogous to results regarding workload (Andreassi, 2000; Cowley et al., 2016). However, pupil diameter is not only dependent on mental states, but also primarily on ambient light conditions. Despite the fact that light conditions in the driving simulator do not change as much as on-road, a correction algorithm was applied by subtracting the *z*-standardized mean pixel luminance from the *z*-values of pupil diameter at every front camera video frame. Even with this adjustment, the effects are still observable. However, this quite simple adjustment procedure has some limitations. First, the exact association between ambient light and pupil diameter is much more complex than a simple linear relationship (Watson and Yellott, 2012). Second, cameras themselves adapt to ambient light, which does not allow to exactly measure luminance out of a video image. Third, eye tracking with the front camera can be used for lab experiments; in automated vehicles, luminance must be measured by other sensors. Despite these limitations, the applied adjustment procedure is real-time capable and will again be tested in subsequent studies within the project.

Eye blink rate showed the expected u-shaped pattern with fewer blinks during the discomfort interval (i.e., participants kept their eyes open in this situation). However, as the baseline blink rate is about one blink every 4 s, eye blinks are a “rare event” in relation to the duration of discomfort intervals. Thus, the low frequency of eye blinks lowers the potential to serve as real-time predictor for discomfort.

Contrary to the expected trend, HR decreased during discomfort periods and returned to the prior level approximately 5 s after reported discomfort. A possible explanation for the unexpected decrease could be the effect of “preparation for action,” which means an anticipatory deceleration of HR prior to planned actions (Schandry, 1998; Cooke et al., 2014). The effect was reported for sport actions such as golf putting, but also for simpler reaction time (RT) paradigms: “It is well established that HR deceleration occurs during the fixed foreperiod of an RT task” (Andreassi, 2000, p. 270). The HRV measured by the RMSSD showed the expected u-shaped pattern with a decrease during the discomfort intervals.

The SCL showed a linear increasing trend over time, which could probably be explained by the effect that participants got warm during driving. After correcting for this linear trend using a regression approach, SCL showed almost no situation-related changes during discomfort intervals. The missing effects could be related to measurement procedures associated with the smartband. First, absolute SCL values were highly dependent on how tightly the band was closed. These differences could be corrected by the *z*-transformation; however, some bias could remain (e.g., when the band was worn very loosely). Second, SCL measures were taken from the outer side of the wrist, which is considered a much less sensitive place for SCL-changes compared with the fingers (Andreassi, 2000). Third, hand movements partly caused strong offsets in EDA values. The simple correction method of excluding these parts from the analysis could potentially be improved by more sophisticated algorithms.

The mentioned problems such as limited control on how tight the band is closed are to some extent related to the use of smartbands instead of more sophisticated measurement devices for physiological parameters. However, the aim of the KomfoPilot project was and is to estimate the potential of existing wearable devices with all the real-world usage challenges. Even with these problems, effects associated with discomfort could be identified in the data. One of the major challenges for using these devices will be the use of adequate signal analysis methods for gaining maximum signal-to-noise ratio.

Body movements captured by the pressure seat mat and the motion tracking of the right shoulder showed the expected pushback during the close approach to the truck. As posture dynamics are strongly related to specific situations (Tran and Trivedi, 2010), these movement patterns cannot automatically be generalized across different discomfort situations. However, discomfort associated with gaps that are too close or potential rear-end collisions could be detected involving body motion. A potential approach for data fusion algorithms could be the inclusion of environment sensor information such as time headway (Leonhardt et al., 2017) and to consider the pushback motion pattern only in these situations.

To sum up, the predicted mechanism of the monitored physiological signals for designing and configuring the real-time detection algorithm includes the following aspects. Most relevant parameters with the highest discomfort-specific changes resulted in ambient light-corrected pupil diameter, HR and the pushback movement. Interblink interval time and HRV measures showed changes, but could be unstable due to the short time intervals. The SCL from the MS Band 2 did not show specific changes and is, therefore, not recommended for inclusion in the algorithm. Regarding variability and filtering, relative changes within one person need to be assessed due to the strong individual component of all parameters. This could be achieved in real-time by e.g., performing individual *z*-standardization in sliding time windows and comparing the current signal value with these scores. This comparison could include several time windows of different lengths and different onsets, e.g., with 10 s and 5 s duration and an onset 3 s and 5 s before the current moment. This procedure would allow one to keep trace of the individual parameter variability by offering, at the same time, the application of standardized thresholds (such as a decrease in HR by 0.3 *SD*-units compared to the sliding window). Threshold values as well as timings can be obtained from the results in Figure 5 and can be adjusted to configure the sensitivity of the detection algorithm. To combine these predictions of each single parameter into one discomfort-score, probabilistic data fusion methods such as Bayesian Networks could be used. The nodes of such a network allow for integration of environment information (such as presence of a vehicle driving ahead) as well as for “inverting” the algorithm, once discomfort was detected, in order to return to the baseline. This method has already been applied by the Communication Engineering Department at Chemnitz University of Technology for real-time prediction of lane change maneuvers, combining parameters from the driver, the vehicle and the environment (Leonhardt et al., 2017).

In conclusion, the assessed parameters from smartbands, eye tracking and motion tracking showed potential for detecting discomfort in this approach situation. Despite commercially available smartbands providing less precise measures as dedicated lab devices, effects associated with discomfort could be identified. However, wearable devices also pose new challenges such as less control on how users apply them. A limitation of this study is of course that only this specific truck approach situation has been investigated. The findings must be validated in other potentially discomfort-inducing situations, which are the next steps in the project. However, the use of this highly standardized approach situation also provides some advantages: (a) a distance that is experienced as too close is one of the most mentioned issues for discomfort as a codriver (dpa, 2013); (b) comfortable adjustment of headway distance and approach situations are not only relevant in conditional and high automation (SAE Levels 3 and above), but also for driver assistance systems such as adaptive cruise control and partial automation (SAE levels 1 and 2); and (c) the high standardization of the situation allowed for estimating the potential of different sensors as well as testing data filtering and artifact-removal strategies. Thus, the results serve as a basis for designing and configuring the real-time detection algorithm that is in development by the project partners who specialize in data fusion (FusionSystems GmbH and Communication Engineering Department at Chemnitz University of Technology). The algorithm will be implemented in the driving simulation software and tested in subsequent studies.

## Author Contributions

MB, FH and JK contributed to conception and design of the study and also contributed to manuscript revision, read and approved the submitted version. MB performed sensor data preparation, setup of the PostgreSQL database and statistical analyses of sensor data. FH preparared and analyzed participant and questionnaire data. MB drafted major parts of “Introduction, Results and Discussion” sections. FH contributed to the “Introduction” section and drafted main parts of the “Materials and Methods” section. JK contributed to the discussion section.

## Conflict of Interest Statement

The authors declare that the research was conducted in the absence of any commercial or financial relationships that could be construed as a potential conflict of interest. The reviewer DW and handling Editor declared their shared affiliation.
